# Composition of peripheral blood immune cell compartment in stage 5 chronic kidney disease is affected by smoking and other risk factors associated with systemic inflammatory response

**DOI:** 10.3389/fimmu.2025.1608206

**Published:** 2025-08-01

**Authors:** Ivan Zahradka, Filip Tichanek, Lucia Majercikova, Jana Duskova, Mathias Streitz, Stephan Schlickeiser, Klara Osickova, Vojtech Petr, Petra Hruba, Petra Reinke, Ondrej Viklicky

**Affiliations:** ^1^ Department of Nephrology, Institute for Clinical and Experimental Medicine, Prague, Czechia; ^2^ Department of Data Science, Institute for Clinical and Experimental Medicine, Prague, Czechia; ^3^ Transplantation Laboratory, Institute for Clinical and Experimental Medicine, Prague, Czechia; ^4^ Department of Experimental Animal Facilities and Biorisk Management, Federal Research Institute for Animal Health, Greifswald-Insel Riems, Germany; ^5^ Berlin Institute of Health Center of Regenerative Therapies (BRCT), Berlin Institute of Health at Charité, Berlin, Germany; ^6^ CheckImmune GmbH, Campus Virchow Klinikum, Berlin, Germany; ^7^ Center for Advanced Therapies (BeCAT), Charité Universitätsmedizin, Berlin, Germany

**Keywords:** peripheral blood immune cell composition, T cells, B cells, dendritic cells, flow cytometry, chronic kidney disease, inflammation

## Abstract

**Background:**

Stage 5 chronic kidney disease (CKD5) is linked to complex yet not fully understood disturbances in immune system. This study aimed to investigate these disturbances by exploring the detailed composition of peripheral blood immune cell compartments in CKD5 patients and to provide integrative, multivariable dissection of how common inflammatory risk factors shape the immune landscape.

**Methods:**

This cross-sectional study included 107 patients with chronic kidney disease stage 5 (CKD5) and 29 healthy blood donors as controls. Peripheral blood B cells, T cells, and dendritic cells were measured using a standardized and validated flow cytometry panel. The impact of selected clinical factors on immune cell composition was initially evaluated using a robust multivariate method (PERMANOVA). Variables that significantly affected immune cell composition were then included in a subsequent series of Poisson regression models, assessing predictors influence on the counts of individual immune cell subpopulations.

**Results:**

Compared to healthy controls, CKD5 patients presented with B cell lymphopenia across all measured subsets except for plasmablasts, T cell lymphopenia with an immunosenescent phenotype predominantly in the CD4^+^ compartment, and significantly higher counts of LIN-HLA-DR^+^ antigen-presenting cells, mainly due to an increase in myeloid dendritic cell subpopulations. PERMANOVA identified smoking, CMV seropositivity, age, dialysis treatment, and atherosclerotic cardiovascular disease as factors significantly influencing peripheral blood immune composition. Subsequent Poisson regression models revealed that smoking was associated mainly with an increase in switched memory B cells, CMV seropositivity with an increase in CD4^+^ and CD8^+^ TEMRA cells, age with a decrease in naive CD8^+^ T cells, and dialysis treatment with an increase in marginal-zone B cells.

**Conclusions:**

Patients with CKD5 exhibit distinct composition of peripheral blood immune cells, further modified by other factors associated with systemic inflammatory response. These factors should be considered in immunomonitoring protocols and may enhance prediction of clinical outcomes such as vaccine responses.

## 1 Introduction

Chronic kidney disease (CKD) impairs both arms of the immune response, with more severe stages of CKD exerting more profound impacts on immune dysfunction (1). As a result, patients with stage 5 CKD (CKD5) have an increased risk of infections (1, 2), rapid progression of atherosclerosis (3, 4), malignancies (5), anemia (6), malnutrition (7), and a poor response to vaccination (8, 9), including new vaccines against COVID-19 (10).

CKD is characterized by chronic, low-grade inflammation (11), which accelerates immunosenescence, particularly within the T cell compartment (12). The hallmarks of the CKD5-associated immunosenescence include a reduction in naive T cells, associated with a reduced efficacy of homeostatic proliferation in the uremic environment (13), and an expansion of memory T-cells driven by lifelong pathogen exposure (14). The increased numbers of memory cells may pose challenges for kidney transplantation as these cells carry immune memory and alloreactivity (15).

Alongside the impacts of CKD itself, other factors associated with systemic inflammatory response may influence the composition of peripheral immune compartments. Some of these factors, such as diabetes, hypertension, obesity or smoking are common among CKD patients and have been shown to affect immune functions. While previous studies have described features of immune dysregulation in CKD5 patients, most have focused selectively on specific immune cell subsets and have not provided a complex, multivariable overview of the immune landscape and have not accounted for the confounding effects of systemic inflammatory factors commonly present in CKD5 patients. While the composition of peripheral blood immune cell compartments is an important reflection of the state of the immune system, and its alterations may be associated with clinical outcomes, there remains a lack of comprehensive, multivariable, standardized characterization of peripheral immune cell subsets in CKD5 patients, especially in a clinically relevant cohort of patients wait-listed for transplantation.

Furthermore, yet another consequence is that systemic inflammatory states may act as previously unaccounted confounding factors in immunomonitoring studies. This poses a major problem, as validated high-quality immunomonitoring remains an unresolved issue in kidney transplantation. Immune response can vary significantly despite the use of similar immunosuppression, which suggests that analyzing immune cell compartment composition may be important.

This study aims to fill the outlined knowledge gap by using a validated flow cytometry panel to analyze peripheral blood immune cell composition in a homogenous cohort of CKD5 patients wait-listed for kidney transplantation. We hypothesized that both immune alterations in CKD5 and other inflammatory factors have a complex combined effect on the landscape of peripheral blood immune cell compartment. We aim to provide a detailed, multivariable dissection of these factors and improve the understanding of immune remodeling in CKD5 to inform future approaches to areas like immunomonitoring, vaccine response assessment or prediction of adverse outcomes.

## 2 Methods

### 2.1 Study design and patient characteristics

This is a single-center, cross-sectional study that enrolled a homogenous cohort of 107 CKD5 patients wait-listed for transplantation that were eventually enrolled in one of 3 prospective studies performed at our center – RIMINI (EudraCT no. 2015-005346-58), CELLIMIN (EudraCT no. 2014-001325-33) and ALIVE (G14-08-38). Blood samples were collected between 1st November 2015 and 20th January 2019 before the transplant surgery.

Control group was formed by 29 healthy blood donors matched for age and sex from the Transfusion Department of Thomayer University Hospital, Prague. Blood donors are considered healthy as they are routinely screened for infectious diseases and regularly fill out health status questionnaires, which rule out chronic diseases (16). However, some potential confounders, such as smoking habits, are not recorded in blood donors and thus are not available for analysis.

The institutional review board approved the individual study protocols under nos. (1619/19, 312/16, 9710/18). Written informed consent was obtained from all participants before blood sample collection.

Electronic medical records were searched for selected clinical and laboratory variables. The selection of variables focused mainly on classic Framingham cardiovascular disease risk factors and factors associated with chronic inflammation as it has been linked to cardiovascular diseases, including past CMV infection (17). Variables selected included age, gender, previous solid organ transplantation, diabetes, smoking, dialysis history, atherosclerotic cardiovascular disease, cause of end stage kidney disease, baseline C-reactive protein (CRP), hepatitis B (HBV) and C (HCV), CMV seropositivity, treated dyslipidemia and hyperuricemia, and body mass index (BMI).

Smoking status was evaluated by the NHIS criteria (18). Atherosclerotic cardiovascular disease was defined as one of the following: history of symptomatic myocardial infarction, cerebrovascular disease, peripheral artery disease or a selective coronarography finding requiring a coronary intervention. CMV, HBV and HCV seropositivity was based on the presence of appropriate IgG antibodies.

### 2.2 Immune cell subtypes definition

Subpopulations of DC, B and T cells were assessed using the previously standardized and validated flow cytometry panel from ONE Study (19). All staining was performed using pre-formulated dry surface antibodies listed in **Supplementary Table 1** according to the manufacture´s protocol as described previously (20) (**Supplementary Material 1**).

T cells are distinguished by the presence of CD3 and can be divided into two distinct subgroups by the presence of either CD4 or CD8 antigen. Based upon the expression of CCR7 and CD45RA, 4 groups of distinct T cells are characterized – naive cells (CCR7^+^CD45RA^+^), effector memory (EM) cells (CCR7^-^CD45RA^-^), central memory (CM) cells (CCR7^+^CD45RA^-^) and terminally differentiated effector memory (TEMRA) cells (CCR7^-^CD45RA^+^). B cells are distinguished by the presence of CD19. Naive B cells express immunoglobulin isotype M and D classes. Based on the expression of IgD and CD27, 2 groups of B cells are characterized – naive (IgD^+^CD27^-^) and marginal-zone B cells (IgD^+^CD27^+^). IgM^+^IgD^+^ cells can be divided into class non-switched B cells (CD27^+^CD38^-^), and from the CD27^-^population transitional B cells (CD27^-^CD24^high^CD38^high^) can be identified. As loss of IgD and IgM expression is a marker of immunoglobulin isotype class switching, pre-gated IgM^-^IgD^-^ cells can further be used to identify class switched memory B cells (CD27^+^CD38^low^) and plasmablasts (CD27^+^CD38^high^). Antigen presenting cells (APCs) are distinguished by expression of HLA-DR antigen. Dendritic cells (DCs) lack the expression of classical mature lymphocyte lineage markers (CD3, CD19, CD20, CD56) and monocytic CD14 (LIN^-^). Dendritic cells are distinguished as LIN^-^ cells with high expression of HLA-DR. Two main subgroups of DCs are present in peripheral blood and can be divided by their expression of CD11c into myeloid DCs (mDCs, CD11c^+^) and plasmacytoid DCs (pDCs, CD11c^-^). Three subsets of mDCs are identified in peripheral blood: mDC1 (CD1c^+^), mDC2 (CLEC9A^+^) and mDC3 (CD16^+^). The gating strategy for T cells, B cells and dendritic cells are summarized in **Supplementary Figures 1**–**3**.

### 2.3 Statistical analysis

Statistical analysis was performed using SPSS version 22.0 (IBM Corp, Armonk, NY, USA) and R version 4.4.1 (R Core Team (2024). R: A language and environment for statistical computing. R Foundation for Statistical Computing, Vienna, Austria. https://www.R-project.org/). As we did not successfully measure the T-lymphocyte counts for one patient, this patient was excluded from all analyses.

#### 2.3.1 Summary statistics and data exploration

Continuous variables are reported as medians with interquartile ranges, and categorical variables as proportions (%). Intergroup differences were calculated with the Mann-Whitney test, chi-square test, or Fischer test as appropriate.

Basic summary statistics revealed that patients with a history of kidney transplantation (n = 3) differed significantly from those without prior transplants. Due to their small sample size and unique characteristics, such as continuing immunosuppression use and previous allosensitization, these patients were excluded from the analyses. No patient with glomerulonephritis as a primary renal disease was treated by immunosuppression in the previous 6 months before obtaining blood samples and therefore no patient was excluded from the analyses for this reason.

#### 2.3.2 Effect of factors associated with systemic inflammatory response

Potential effect of inflammatory factor on immune cells counts and composition were assessed in the following three steps.

First, we performed single-variable Permutational Analyses of Variance (PERMANOVAs) using the ‘Vegan’ R package (21) to identify which factors explain the largest portion of variance in the multivariate peripheral blood cell composition when considered as single predictors. We then selected those that explained more than 2% of variance of outcome data with (R^2^ > 0.02) and included them in a multivariable PERMANOVA, using sequential variance partitioning and ordered by R^2^ (starting with the most influential factor). This analysis was conducted for all measured peripheral blood immune cell subpopulations together (‘joint PERMANOVA’) as well as separately for subsets of specific data of similar cell types (DC, B cells, T cells). All PERMANOVAs were run with Euclidean distances after log10-transformation and z-standardization of the cell counts, using 5000 permutations. Predictors (clinical characteristics) that showed a statistically significant effect in any of the multivariable PERMANOVAs (joint, DC, B, T cells) were then used in a series of generalized linear models, fitted with ‘glmmTMB’ package (22). These models predicted the count of a specific sub-populations assuming a Poisson distribution, with observation-specific random intercepts to account for overdispersion. P-values were corrected using the Benjamini-Hochberg correction (FDR) for multiple comparisons (23), considering 18 models with the same set of predictors but different outcomes (individual immune cell subpopulations). The results were visualized using a volcano plot, showing the estimated effect of the predictor on the X axis (as estimated log-fold difference in count when the predictor value increases by 1 unit) and the -log10(P-value) on the Y axis (the higher the value, the stronger the evidence of the effect direction). Furthermore, we also show Spearman’s rank correlation between peripheral immune cell compartment sub-populations and the selected patients characteristics, visualized with ‘ComplexHeatmap’ R package (24). For the visualization, sub-populations were clustered based on similarity in distribution of their abundance using unweighted pair group method with arithmetic mean (UPGMA). All tests were performed at the 5% level of significance. The detailed statistical report as well as raw anonymized data can be found online in the GitHub repository (25).

## 3 Results

### 3.1 Peripheral blood immune cell compartment changes in CKD5 patients compared to healthy blood donors

The demographic data of the CKD patients are summarized in **Table 1**; the characteristics shown divided according to smoking, dialysis, CMV seropositivity and presence of atherosclerotic cardiovascular disease status are shown in **Supplementary Table 2**. Firstly, B cells, T cells, and DCs subsets were compared in the CKD5 and the control healthy blood-donor control group. The results are summarized in **Table 2**.

**Table 1:** Basic characteristics of patients in the CKD group.

Basic characteristics – CKD patients	
number of subjects	107
age (years), median (IQR)	50.8 (41.76 – 62.18)
female sex, n (%)	31 (29%)
diabetes status, n (%)	23 (21.5%)
BMI, mean (range)	27.6 (16.1 – 39.8)
current smoker, n (%)	29 (27.1%)
ASCVD, n (%)	21 (19.6%)
dialysis treatment, n (%)	80 (74.8%)
dialysis vintage (months), median (IQR)	19.5 (8.63 – 37.04)
CMV seropositivity, n (%)	87 (81.3%)
treated hyperuricemia, n (%)	45 (42.1%)
treated dyslipidemia, n (%)	55 (51.4%)
glomerulonephritis, n (%)	49 (45.8%)
albumin, median (IQR)	41.5 (38.3 - 44)
C-reactive protein, median (IQR)	2.5 (1 – 5.5)
White blood cell count (10^9^/l), median (IQR)	8 (6.4 - 9.6)

ASCVD, atherosclerotic cardiovascular disease; BMI, body mass index; CMV, cytomegalovirus.

**Table 2:** Comparisons of immune cell subpopulations in CKD and healthy controls.

Comparison of cell subpopulations in CKD5 and healthy controls				
Cell subpopulation	CKD5 group (median; IQR) [cells/μL]	Healthy controls (median; IQR) [cells/μL]	p-value	Mean counts ratio
Dendritic cells				
HLA-DR+ cells	60.23 (50.88)	45.83 (55.18)	0.046	1.31
total mDC	47.5 (44.46)	26.52 (62.37)	<0.001	1.79
mDC1	8.04 (6.81)	5.91 (7.33)	0.015	1.57
mDC2	0.33 (0.46)	0.3 (0.31)	0.3	0.92
mDC3	35.49 (38.72)	18.43 (44.13)	0.001	1.79
pDC	7.99 (8.65)	8.23 (9.41)	0.97	1.15
B cells				
total CD19+ B cells	82.2 (80.67)	213.31 (115.95)	<0.001	0.45
naive B cells	44.56 (53.35)	119.53 (85.61)	<0.001	0.41
marginal-zone B cells	11.2 (15.24)	30.48 (24.23)	<0.001	0.44
class non-switched memory cells	9.24 (16.77)	29.38 (17.55)	<0.001	0.44
class switched memory cells	8.74 (12.53)	18.38 (20.01)	<0.001	0.56
plasmablasts	0.39 (0.67)	0.46 (0.47)	0.6	1.78
transitional B cells	6.1 (9.02)	10.36 (11.74)	0.003	0.55
T cells				
total CD3+ cells	1036.86 (769.86)	1517.1 (611.67)	0.001	0.8
total CD4+ cells	689.24 (435.37)	820.94 (358.93)	0.043	0.9
CD4+ CM cells	333.86 (268.81)	377.55 (162.56)	0.3	0.98
CD4+ EM cells	88.39 (85.73)	52.98 (81.54)	0.006	1.59
CD4+ naive cells	183.54 (190.93)	309.59 (168.53)	<0.001	0.64
CD4+ TEMRA cells	5.43 (13.51)	7.04 (13.46)	0.4	1.67
total CD8+ cells	326.15 (316.57)	467.43 (353.46)	0.006	0.72
CD8+ CM cells	41.19 (49.39)	53.72 (34.19)	0.2	0.94
CD8+ EM cells	67.98 (78.65)	124.48 (122.48)	0.003	0.63
CD8+ naive cells	68.61 (76.86)	97.68 (68.89)	0.13	0.78
CD8+ TEMRA cells	107.81 (138.95)	149.99 (209.14)	0.085	0.69

Absolute numbers (cell/μl), distribution is shown as median (IQR).

When compared to the control group, CKD5 patients presented with significant B cell lymphopenia, both in total B cells and in all the measured subsets, except for plasmablasts. CKD5 patients also presented with a significant reduction in both total CD3^+^ T cell, and CD4^+^ and CD8^+^ T cell subsets. In more depth, a significant reduction in the count of CD4^+^ naive and CD8^+^ EM, and an increase in CD4^+^ EM cells were found. Other subsets of T-cells did not differ between the groups.

Finally, CKD5 patients had significantly higher counts of LIN^-^HLA-DR^+^ APCs. Further analysis of mDC subpopulations revealed a marked increase in mDC1 and mDC3 dendritic cells but there was no clear difference in pDCs.

### 3.2 Factors associated with variance of peripheral blood cell composition

We examined the factors explaining the variance in peripheral blood cell compartment in CKD5 patients using the PERMANOVA method. First, single-predictor PERMANOVAs were calculated with all selected clinical variables. These analyses were performed across all measured immune cell subsets and separately for B cells, T cell and DC subsets. The results are shown in **Supplementary Tables 3**–**6**.

Next, variables from single-predictor PERMANOVAs were selected based on the value of R^2^ and multivariable PERMANOVA was calculated, again across all immune cell subsets and separately B cell, T cell, and DCs subsets. Results are summarized in **Table 3**. We found that smoking (R2 = 0.0405, p < 0.001) and CMV seropositivity (R2 = 0.0227, p = 0.02) were significantly associated with the composition of peripheral blood immune cell compartment overall. When analyzed separately, smoking was the only factor strongly associated with the variance in all three cell subsets – DCs (R2 = 0.0282, p = 0.03), B cells (R2 = 0.0447, p = 0.008), and T cells (0.0392, p = 0.003). Furthermore, dialysis was associated with changes in the composition of B cell compartment (R2 = 0.0352, p = 0.019), while CMV seropositivity (R2 = 0.0504, p < 0.001), age (R2 = 0.333, p = 0.008), and presence of ASCVD (R2 = 0.0237, p = 0.035) were associated with changes in the T cell compartment. Furthermore, a sensitivity analysis where factors into multivariable PERMANOVAs were selected based on p < 0.05. The results of this sensitivity analysis were consistent with the results of the primary analysis (**Supplementary Table 7**).

**Table 3:** Results of multivariable PERMANOVAs calculated across different peripheral blood immune cell subsets.

Multivariable PERMANOVA across all cell subsets		
Characteristic	R2	p-value
smoking	0.0405	<0.001
CMV seropositivity	0.0227	0.02
ASCVD	0.018	0.06
diabetes mellitus	0.0134	0.15
age	0.02	0.03
Multivariable PERMANOVA across dendritic cell subsets		
Characteristic	R2	p-value
smoking	0.0282	0.03
ASCVD	0.0233	0.06
Multivariable PERMANOVA across B-cell subsets		
Characteristic	R2	p-value
smoking	0.0447	0.008
dialysis	0.0352	0.019
diabetes mellitus	0.0171	0.13
ASCVD	0.0065	0.51
hyperuricemia	0.0053	0.61
Multivariable PERMANOVA across T-cell subsets		
Characteristic	R2	p-value
CMV seropositivity	0.0504	<0.001
smoking	0.0392	0.003
age	0.0333	0.008
ASCVD	0.0237	0.035
diabetes mellitus	0.01	0.32

**Figure 1:**
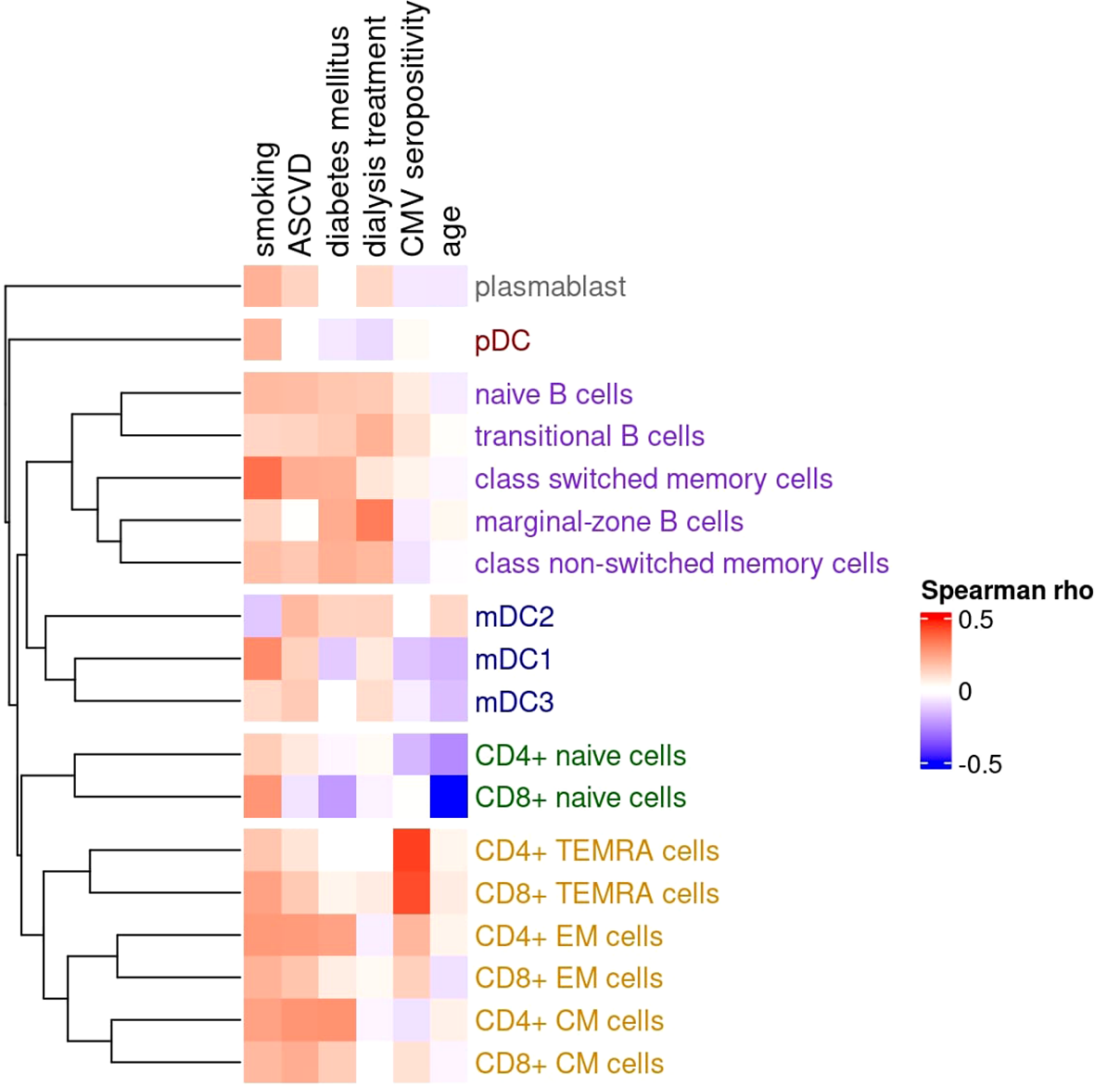
Matrix of Spearman correlation coefficients between clinical characteristics and counts of various peripheral blood immune cell subpopulations. The dendrogram illustrates similarities in cell counts, where closely linked tips indicate that the associated immune cell subpopulations are interrelated, with higher counts tending to co-occur. Correlations with a cluster of related co-occurring sub-populations suggest a specific influence (e.g., CMV infection), while correlations with individual subpopulations across different clusters indicate a more diffuse effect (e.g., smoking). The dendrogram is constructed using the unweighted pair group method with arithmetic mean (UPGMA) clustering. Clusters of subpopulations (as defined by UPGMA) are as follows: (1, grey) circulating plasmablast; (2, red) plasmacytoid dendritic cells (pDC); (3, purple) B cell compartment – including naive B cells, transitional B cells, class-switched memory cells, marginal-zone B cells, and class non-switched memory cells; (4, blue) myeloid dendritic cells (mDC1, mDC2, mDC3); (5, green) naive T cell compartment – CD4+ and CD8+ naive T cells; and (6, orange) memory T cell compartment – including CD4+ and CD8+ TEMRA, effector memory (EM), and central memory (CM) subsets.

The correlations between selected clinical characteristics and peripheral blood immune cell subsets are shown in **Figure 1**.

### 3.3 Associations between specific immune cell subsets and factors associated with systemic inflammatory response

**Figure 2:**
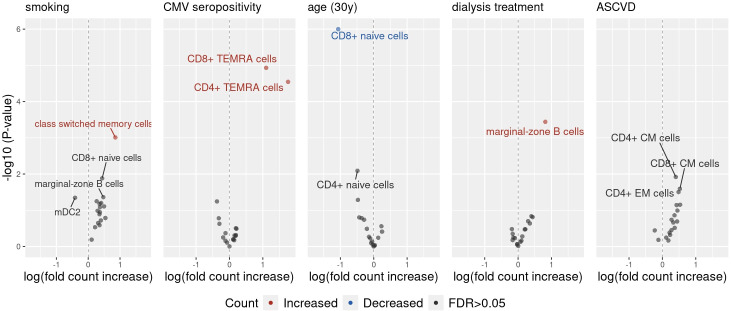
The volcano plot illustrates the effects of clinical characteristics on the counts of various peripheral blood immune cell subpopulations, estimated using a generalized linear model (GLM) with a Poisson distribution. The x-axis represents the effect size of each predictor on the outcomes. For example, an effect size of 0.85 for smoking on switched memory cells suggests that smoking is associated with a 2.33-fold increase in the count of these cells, as determined by exp(0.85). The y-axis displays the −log10(P-value) for each association, before P-value adjustment. Only associations with unadjusted P-values below 0.05 are labelled.

Next, the association between selected factors and specific immune cell subsets was evaluated. Clinical characteristics that showed statistically significant associations with the composition of peripheral blood immune cell compartments in previously performed multivariable PERMANOVAs were studied. A series of generalized linear models was performed (**Supplementary Table 8**) and the results were visualized by volcano plots (**Figure 2**).

Smoking was most strongly associated with an increase in the count of switched memory B-cells . The presence of anti-CMV IgG antibodies was associated with an increase in both CD4 ^+^ and CD8 ^+^ TEMRA T-cells . Age was associated with decrease in naive CD8 ^+^ T-cells, but not in naive CD4 ^+^ or naive B-cells. Lastly, dialysis was associated with an increase in the count of marginal-zone B-cells, while the presence of ASCVD was not significantly associated with the change of any single peripheral blood immune cell count. Furthermore, a sensitivity analysis with a linear model with log-transformed outcomes was performed and yielded consistent results with the primary model (**Supplementary Table 9**).

## 4 Discussion

In this cross-sectional study, we evaluated the patterns of peripheral blood immune cell compartment composition in patients with CKD5 and other metabolic and non-metabolic conditions associated with systemic inflammatory response. We found distinct changes in CKD5 patients, the most apparent being severe B cell lymphopenia and pronounced immunosenescent phenotype in T cell subsets. Furthermore, several additional factors linked to complex changes in the peripheral blood immune cell compartments were identified with smoking being the strongest factor, followed by age, dialysis treatment, CMV seropositivity, and ASCVD.

A significant decrease of both total and all studied subsets of B cells was found in patients with CKD5, except for plasmablasts. This is in line with two previous studies that reported similar results (26, 27). Decreased numbers of total B cells and their subtypes might play a role in the mechanism of non-responsiveness to vaccination, which has been observed in CKD5 patients for several vaccines including hepatitis B (8) or pneumococcal vaccines (9). Vaccine non-responsiveness has become an even greater issue during the COVID-19 pandemic as both patients on chronic maintenance dialysis and after kidney transplantation are reported to have decreased response to SARS-CoV-2 vaccines (10, 28–30). The pathogenesis of vaccine non-responsiveness is not yet fully understood, but one of the factors linked to poor vaccine response is low total CD19^+^ B cell count (31, 32) and low naive B cell count (33) in various non-CKD related diseases. This is further supported by the observation that B-cell depleting agent rituximab is also associated with poor vaccine response (34). This suggests, that although the cooperation of B cells, dendritic cells and helper T cells is expected for normal antibody response, the marked decrease of almost all B cell subtypes in patients with CKD5 is a major factor behind vaccine non-responsiveness. Interestingly, the degree of depletion of immature B cells might also influence kidney transplantation outcomes as it was previously reported that low levels of transitional B cells are linked to higher risk of allograft rejection (35). This shows that these changes in the B cell compartment, manifested by B cell lymphopenia especially of immature cells, can be associated with a complex immune dysregulation possibly leading to the increased alloreactivity on one hand, and the poor vaccination response on the other.

The findings of CD4^+^ T cell alterations and immunosenescent phenotype are in line with other published studies such as the study by Chiu et al. (12). However, there are some differences in the CD8^+^ T cell subset, which can be likely attributed to different study populations. Unlike Chiu et al. who included a broad dialysis population, we included only patients wait-listed for kidney transplantation that tend to be younger and have fewer comorbidities. Differences in the biological age may explain diverse findings within the CD8^+^ T cells subsets. In a normal healthy population, the hallmark of ageing of adaptive immune system is the involution of thymus and subsequently decreased output of naive T cells (36). Afterwards, the naive CD4^+^ T cell population is maintained by a homeostatic proliferation (37). This mechanism is, however, less effective in CKD where a pro-apoptotic profile of T cells was described (13). While CD8^+^ naive cells are maintained similarly, it is to a lesser extent, which causes faster decrease with age (38). This may explain why the CD4^+^ compartment was comparable, but patients in our cohort showed less immunosenescent phenotype in the CD8^+^ cell compartment than those in study by Chiu et al.

We found that CMV seropositivity was strongly associated with changes in the T cell compartment, mainly increase in both CD4^+^ and CD8^+^ TEMRA cells. The association between CMV latency and accelerated immunosenescence accompanied by the expansion of highly differentiated memory T cells in subjects without CKD has been established well and we confirm this pattern in CKD5 patients (39). This association has a potential impact in kidney transplantation and several studies have reported the link between TEMRA cells and increased risk of rejection and graft failure. Ngoc et al. showed that humoral rejection is associated with accumulation of cytolytic CD8^+^ TEMRA cells both in peripheral blood and kidney graft biopsies and that these cells exhibited enhanced migratory properties (40). Additionally, Vaulet et al. showed strong association between CD8^+^ TEMRA cells and graft failure, regardless of the rejection histology phenotypes (41). Furthermore, a link between liver graft rejection and high levels of CD4^+^ TEMRA cells has also been reported (42). One of the possible mechanisms explaining this phenomenon is the induction of cross-reactive T cells to alloantigens by CMV infection (43).

This study also confirms our previous report (20) of a link between chronic dialysis treatment and augmentation of marginal zone B cell population on a larger patient cohort. Marginal-zone B cells are involved in the early antibody response (44) and produce low affinity, polyreactive IgM antibodies (45, 46). Furthermore, as they have a memory phenotype, repeated antigen exposure leads to rapid activation (47, 48). Dialysis inevitably leads to repeated exposure to bacterial and other antigens and thus augmentation of marginal-zone B cells. As dialysis treatment and dialysis vintage are associated with allosensitization and inferior transplantation outcomes and graft survival (49), marginal-zone B cells could be one of the factors linking dialysis to inferior transplantation outcomes. In fact, a recent mechanistic study has shown that marginal-zone B cells play an essential role in humoral response to kidney and heart allografts and DSA formation (50). The authors has shown that upon antigen stimulation, marginal-zone B cells can rapidly differentiate into antibody-secreting cells in response to transplantation and they can be sufficient in maintaining T-dependent IgM and early IgG DSA production despite depletion of follicular B cells. Furthermore, presence of marginal-zone B cells is required to support the generation of isotype-switched DSA in grafts containing donor-reactive memory helper T cells while recipients lacking marginal-zone B cells have impaired humoral response and DSA formation.

Smoking has been found to have a strong and complex impact on the composition of peripheral blood immune cell compartments. In fact, it was the only significant factor across all studied cell subsets, which well demonstrates the disruptive action smoking has on the immune system. In CKD, smoking is associated with faster progression and, furthermore, with higher risk of graft failure after kidney transplantation (51). Whether this can be explained simply by faster progression of cardiovascular disease, or if an immunological component is at play, is currently unknown. In our study, the strongest association was found between smoking and increased levels of class-switched memory B cells, which have previously also been linked to development of COPD pathogenesis (52). Smoking is linked to chronic antigen exposure and immune remodeling which in turn promotes B cell activation and germinal center formation in lungs. As B cells are known to traffic to the circulation after antigen recognition in the lung, the augmentation of class-switched memory B cells in peripheral blood might be a reflection of B-cell response in the lungs. This theory is further supported by the fact that the increased class switched memory B cell population consists mainly of IgA expressing B cells. Considering the disruptive action smoking has on the immune system as well as its association with poor transplantation outcomes, it is clear that programs aimed at smoking cessation in wait-listed patients should be established and encouraged (53).

One of the major advantages of our study is the use of a standardized and well-defined immune monitoring panel based on the One Study (19) and BIO-DrIM clinical trials (Rimini, Cellimin). Results acquired by these procedures allow for a meaningful comparison of immune cell monitoring panels between different centers and across different trials.

Among the limitations of this study is that the study cohort included participants of three different interventional and observational studies. This does not hinder the evaluation of adaptive immune cells in peripheral blood pre-transplant but precludes a meaningful patient follow-up and evaluation of longitudinal outcomes. The cross-sectional nature of the study precludes causal inference. Among the other limitations is that our study population is representative of wait-listed patient population and therefore can differ from cohorts made up of unselected dialyzed patients. Furthermore, the sample size of the control group is modest and, despite recruiting the control group from a blood donor program, which requires absence of almost all comorbidities, we have no data on some factors such as smoking status. Additionally, latent or unmeasured confounders, such as previous use of immunosuppressives or nutritional status, could influence the results. Lastly, no functional assays were used in this study.

In conclusion, this study provides a comprehensive and standardized analysis of peripheral immune cell composition in a well-defined cohort of CKD5 patients wait-listed for transplantation. We identified consistent patterns of immune dysregulation associated with CKD, most notably B cell lymphopenia and T cell immunosenescence, along with the modifying effects of systemic inflammatory factors such as smoking, CMV seropositivity or history of dialysis. These findings contribute to a more complex understanding of immune system remodeling in CKD5. Investigators running kidney transplantation studies should be aware of the impact of inflammatory factors on the immune landscape and take them into account when analyzing immunomonitoring data. Although some of the findings have potential clinical impact, it needs to be explored further whether these immune alterations can help predict adverse outcomes or whether specific cell subtypes could be appropriate therapeutic targets.

## Data Availability

The raw data supporting the conclusions of this article will be made available by the authors, without undue reservation.
