# Supplementary material for: Composition of peripheral blood immune cell compartment in stage 5 chronic kidney disease is affected by smoking and other risk factors associated with systemic inflammatory response
Source: Front Immunol. 2025 Aug 1;16:1608206. doi: 10.3389/fimmu.2025.1608206 (PMC12353689; doi:10.3389/fimmu.2025.1608206)
Supplement: Supplementary file 1 [file Table1.docx]

Supplementary Materials

# Supplementary Material 1. Blood sample collection and flow cytometry staining protocol

Subpopulations of DC, B and T cells were assessed using the previously standardized and validated flow cytometry panel from ONE Study. Blood was collected into test tubes with ethylenediaminetetraacetic acid as anticoagulant. Cell staining procedure was performed within 4 hours of blood sample collection, for B cell staining the whole blood was washed twice with phosphate-buffered saline (PBS). All staining was performed using pre-formulated dry surface antibodies (DURAClone IM B cells (B53318), IM T cells (B53328) and IM DC cells (B53351); Beckman Coulter) according to the manufacture´s protocol. DURAClone IM Count Tube: CD45-FITC, 7-AAD (clone J33) and fluorescent beads were used to determine the absolute numbers of each of the sub-populations. Samples were then analyzed by the Navios flow cytometer (Beckman Coulter), the data were analyzed with Kaluza software (Beckman Coulter).

|  | **Fluorochrome** | **FITC** | **PE** | **ECD** | **PC5.5** | **PC7** | **APC** | **A700** | **APC-A750** | **Pacific Blue** | **Krome Orange** |
| --- | --- | --- | --- | --- | --- | --- | --- | --- | --- | --- | --- |
| **T panel** | Antibody | CD45RA | CD197/CCR7 | CD28 | CD297/PD1 | CD27 | CD4 | CD8 | CD3 | CD57 | CD45 |
|  | Clone | 2H4 | G043H | CD28.2 | PD1.3.5 | 1A4.CD27 | 13B8.2 | B9.11 | UCHT-1 | NC1 | J33 |
| **B panel** | Antibody | IgD | CD21 | CD19 |  | CD27 | CD24 |  | CD38 | IgM | CD45 |
|  | Clone | IA6–2 | BL13 | J3–119 |  | 1A4CD27 | ALB9 |  | LS198–4-3 | SA-DA4 | J33 |
| **DC panel** | Antibody | CD16 | CD3 |  | CD1c | CD11c | Clec9A | CD123 |  | HLADR | CD45 |
|  | Clone | 3G8 | UCHT-1 |  | L161 | BU15 | 8F9 | SSDCLY107D2 |  | IMMU-357 | J33 |
|  | Antibody |  | CD14 |  |  |  |  |  |  |  |  |
|  | Clone |  | RMO52 |  |  |  |  |  |  |  |  |
|  | Antibody |  | CD19 |  |  |  |  |  |  |  |  |
|  | Clone |  | J3-119 |  |  |  |  |  |  |  |  |
|  | Antibody |  | CD20 |  |  |  |  |  |  |  |  |
|  | Clone |  | HRC20 |  |  |  |  |  |  |  |  |
|  | Antibody |  | CD56 |  |  |  |  |  |  |  |  |
|  | Clone |  | N901 |  |  |  |  |  |  |  |  |

# Supplementary Table 1. DuraClone IM T, B and DC flow cytometry panels.

**Supplementary Table 2. Basic characteristics of the study group divided by smoking, dialysis, CMV seropositivity and ASVD status.**

ASCVD (atherosclerotic cardiovascular disease), BMI (body mass index), CMV (cytomegalovirus)

| **Basic characteristics** | | | | | | | | | | | | |
| --- | --- | --- | --- | --- | --- | --- | --- | --- | --- | --- | --- | --- |
| **Characteristic** | **Smoking** | | | **Dialysis** | | | **CMV seropositivity** | | | **ASCVD** | | |
|  | **yes**  **(n = 29)** | **no**  **(n = 78)** | **p** | **yes**  **(n = 80)** | **no**  **(n = 27)** | **p** | **yes**  **(n = 87)** | **no**  **(n = 20)** | **p** | **yes**  **(n = 21)** | **no**  **(n = 86)** | **p** |
| age (years), median (IQR) | 52.1  (38.2 – 61.3) | 53.8  (43 – 62.4) | 0.664 | 53.6  (42.4 – 62.4) | 53  (37.7 – 62) | 0.866 | 53.5  (43 – 62.4) | 47.1  (35.3 – 60.7) | 0.255 | 58.3  (53.5 – 64) | 51.2  (37.6 – 59.7) | 0.003 |
| female sex, n (%) | 5 (17.2) | 26 (33.3) | 0.103 | 20 (25) | 11 (40.7) | 0.119 | 27 (31) | 4 (20) | 0.327 | 2 (9.5) | 29 (33.7) | 0.028 |
| diabetes status, n (%) | 8 (27.6) | 15 (19.2) | 0.350 | 19 (23.8) | 4 (14.8) | 0.328 | 20 (23) | 3 (15) | 0.555 | 12 (57.1) | 11 (12.8) | <0.001 |
| BMI, median (IQR) | 28.1  (24.6 – 31.1) | 27.4  (23.6 – 30.6) | 0.430 | 27.6  (24.2 – 31.5) | 27.7  (23.6 – 30.6) | 0.747 | 27.7  (24.2 – 31.2) | 27.3  (23.5 – 29.3) | 0.204 | 30.6  (27.2 – 32.9) | 27.1  (23.4 – 30.1) | 0.009 |
| current smoker, n (%) | N/A | | | 22 (27.5) | 7 (25.9) | 0.874 | 26 (29.9) | 3 (15) | 0.177 | 10 (47.6) | 19 (22.1) | 0.018 |
| ASCVD, n (%) | 10 (34.5) | 11 (14.1) | 0.018 | 18 (22.5) | 3 (11.1) | 0.198 | 18 (20.7) | 3 (15) | 0.758 | N/A | | |
| dialysis, n (%) | 22 (75.9) | 58 (74.4) | 0.874 | N/A | | | 67 (77) | 13 (65) | 0.265 | 18 (85.7) | 62 (72.1) | 0.198 |
| dialysis vintage (months), median (IQR) | 9.7  (1.6 – 40.5) | 11.3  (0 – 26.2) | 0.606 |  |  |  | 11.3  (0.8 – 32) | 10.1  (0 – 31.1) | 0.576 | 24  (15 – 41.9) | 8.5  (0 – 25.9) | 0.008 |
| CMV seropos., n (%) | 26 (89.7) | 61 (78.2) | 0.177 | 67 (83.8) | 20 (74.1) | 0.265 | N/A | | | 18 (85.7) | 69 (80.2) | 0.758 |
| hyperuricemia, n (%) | 10 (34.5) | 35 (44.9) | 0.333 | 24 (30) | 21 (77.8) | <0.001 | 34 (39.1) | 11 (55) | 0.193 | 7 (33.3) | 38 (44.2) | 0.366 |
| dyslipidaemia, n (%) | 17 (58.6) | 38 (48.7) | 0.362 | 42 (52.3) | 13 (48.2) | 0.696 | 47 (54) | 8 (40) | 0.258 | 17 (81) | 38 (44.2) | 0.003 |
| glomerulonephritis, n (%) | 14 (48.3) | 35 (44.9) | 0.665 | 37 (46.3) | 12 (44.4) | 0.960 | 39 (44.8) | 10 (50) | 0.608 | 5 (23.8) | 44 (51.2) | 0.031 |
| albumin, median (IQR) | 41.5  (38 – 43.8) | 41.6  (38.4 – 44) | 0.861 | 42.1  (38.5 – 44.1) | 41  (37.9 – 43.6) | 0.184 | 41.7  (38.7 – 44) | 41.3  (36.8 – 43.6) | 0.322 | 42.5  (40.4 – 44) | 41.3  (38.2 – 44) | 0.153 |
| C-reactive protein, median (IQR) | 2  (0.9 – 8.4) | 2.6  (1.3 – 5) | 0.785 | 2.6  (1.2 – 11.5) | 2.2  (0.8 – 3.5) | 0.200 | 2.3  (1 – 4.6) | 3.7  (1.3 – 7.2) | 0.417 | 1.7  (0.8 – 6) | 2.5  (1.2 – 5.4) | 0.510 |
| White blood cell count (10^9^/l), median (IQR) | 9.8  (7.7 – 11.4) | 7.3  (6.2 – 9.3) | 0.003 | 8  (6.2 – 9.5) | 7.2  (6.6 – 10.1) | 0.722 | 8  (6.3 – 9.5) | 7.7  (6.5 – 10.6) | 0.678 | 8.8  (7.1 – 10.3) | 7.7  (6.2 – 9.5) | 0.208 |

**Supplementary Table 3. Results of single-predictor PERMANOVAs performed across all measured peripheral blood immune cell subsets.**

| **Single-predictor PERMANOVAs across all cell subsets** | | |
| --- | --- | --- |
| **Characteristic** | **R2** | **p-value** |
| smoking | 0.0405 | 0.001 |
| ASCVD | 0.0268 | 0.01 |
| CMV seropositivity | 0.0247 | 0.018 |
| diabetes mellitus | 0.0219 | 0.03 |
| age | 0.0202 | 0.042 |
| dialysis | 0.0148 | 0.13 |
| dyslipidemia | 0.0129 | 0.21 |
| presence of anti-HLA Ab | 0.0113 | 0.29 |
| female sex | 0.0111 | 0.32 |
| hyperuricemia | 0.0094 | 0.4 |
| glomerulonephritis | 0.0062 | 0.78 |
| hepatitis B | 0.0052 | 0.79 |
| hypertension | 0.0031 | 0.97 |

**Supplementary Table 4. Results of single-predictor PERMANOVAs performed across B-cell peripheral blood subsets.**

| **Single-predictor PERMANOVAs across B-cell subsets** | | |
| --- | --- | --- |
| **Characteristic** | **R2** | **p-value** |
| smoking | 0.0447 | 0.008 |
| dialysis | 0.0354 | 0.023 |
| diabetes mellitus | 0.0277 | 0.041 |
| ASCVD | 0.0213 | 0.093 |
| hyperuricemia | 0.0203 | 0.098 |
| presence of anti-HLA Ab | 0.019 | 0.11 |
| female sex | 0.0171 | 0.15 |
| dyslipidemia | 0.0071 | 0.52 |
| CMV seropositivity | 0.0046 | 0.71 |
| glomerulonephritis | 0.0045 | 0.72 |
| hepatitis B | 0.0039 | 0.73 |
| hypertension | 0.0029 | 0.84 |
| age | 0.0026 | 0.89 |

**Supplementary Table 5. Results of single-predictor PERMANOVAs performed across T-cell peripheral blood subsets.**

| **Single-predictor PERMANOVAs across T-cell subsets** | | |
| --- | --- | --- |
| **Characteristic** | **R2** | **p-value** |
| CMV seropositivity | 0.0504 | 0.001 |
| smoking | 0.0437 | 0.002 |
| age | 0.036 | 0.008 |
| ASCVD | 0.0331 | 0.011 |
| diabetes mellitus | 0.0242 | 0.05 |
| dyslipidemia | 0.019 | 0.098 |
| presence of anti-HLA Ab | 0.0103 | 0.36 |
| hepatitis B | 0.0074 | 0.48 |
| female sex | 0.0064 | 0.63 |
| glomerulonephritis | 0.0063 | 0.64 |
| hyperuricemia | 0.004 | 0.84 |
| hypertension | 0.0031 | 0.86 |
| dialysis | 0.0013 | 0.99 |

**Supplementary Table 6. Results of single-predictor PERMANOVAs performed across dendritic cell peripheral blood subsets.**

| **Single-predictor PERMANOVAs across dendritic cell subsets** | | |
| --- | --- | --- |
| **Characteristic** | **R2** | **p-value** |
| smoking | 0.0282 | 0.034 |
| ASCVD | 0.0225 | 0.068 |
| age | 0.0154 | 0.18 |
| female sex | 0.0113 | 0.32 |
| dialysis | 0.0107 | 0.33 |
| dyslipidemia | 0.0096 | 0.41 |
| diabetes mellitus | 0.0087 | 0.44 |
| glomerulonephritis | 0.0087 | 0.45 |
| CMV seropositivity | 0.004 | 0.77 |
| hyperuricemia | 0.0037 | 0.81 |
| hypertension | 0.0035 | 0.8 |
| hepatitis B | 0.0027 | 0.85 |
| presence of anti-HLA Ab | 0.0019 | 0.93 |

**Supplementary Table 7. Sensitivity analysis of multivariable PERMANOVAs.** The variables into multivariable analyses were selected based on p < 0.05, rather than R2 > 0.02. The results are consistent with the results of the primary analysis.

| **Multivariable PERMANOVA across all cell subsets** | | |
| --- | --- | --- |
| **Characteristic** | **R2** | **p-value** |
| smoking | 0.0405 | <0.001 |
| CMV seropositivity | 0.0227 | 0.02 |
| ASCVD | 0.018 | 0.06 |
| diabetes mellitus | 0.0134 | 0.15 |
| age | 0.02 | 0.03 |
| **Multivariable PERMANOVA across dendritic cell subsets** | | |
| **Characteristic** | **R2** | **p-value** |
| smoking | 0.0282 | 0.03 |
| **Multivariable PERMANOVA across B-cell subsets** | | |
| **Characteristic** | **R2** | **p-value** |
| smoking | 0.0447 | 0.008 |
| dialysis | 0.0351 | 0.017 |
| diabetes mellitus | 0.0170 | 0.13 |
| **Multivariable PERMANOVA across T-cell subsets** | | |
| **Characteristic** | **R2** | **p-value** |
| CMV seropositivity | 0.0504 | <0.001 |
| smoking | 0.0392 | 0.003 |
| age | 0.0333 | 0.008 |
| ASCVD | 0.0237 | 0.035 |
| diabetes mellitus | 0.01 | 0.32 |

**Supplementary Table 8. Results of generalized linear models showing the association between clinical variables and the composition of peripheral blood immune cell subsets**. The `CR` suffix indicates the count ratio, representing the expected fold change in the outcome when the predictor increases by one unit. Adjusted P-values are were corrected for multiple testing using the Benjamini-Hochberg correction, which accounts for the repeated estimation of each predictor’s effect across 18 outcomes.

| **Outcome** | **Smoking** | | | **CMV seropositivity** | | | **age (scale of 30 years)** | | | **dialysis** | | | **ASCVD** | | |
| --- | --- | --- | --- | --- | --- | --- | --- | --- | --- | --- | --- | --- | --- | --- | --- |
|  | **CR** | **p** | **adj. p** | **CR** | **P** | **adj. p** | **CR** | **p** | **adj. p** | **CR** | **p** | **adj. p** | **CR** | **p** | **adj. p** |
| mDC1 | 1.45 | 0.082 | 0.164 | 0.73 | 0.167 | 0.751 | 0.75 | 0.183 | 0.550 | 1.22 | 0.337 | 0.759 | 1.44 | 0.138 | 0.355 |
| mDC2 | 0.66 | 0.045 | 0.164 | 1.00 | 0.997 | 0.997 | 1.26 | 0.277 | 0.712 | 1.14 | 0.527 | 0.880 | 1.53 | 0.072 | 0.259 |
| mDC3 | 1.12 | 0.650 | 0.650 | 0.93 | 0.786 | 0.832 | 0.62 | 0.052 | 0.312 | 1.35 | 0.200 | 0.759 | 1.57 | 0.102 | 0.307 |
| pDC | 1.50 | 0.063 | 0.164 | 1.12 | 0.644 | 0.783 | 1.02 | 0.941 | 0.997 | 0.85 | 0.448 | 0.880 | 0.80 | 0.361 | 0.499 |
| naive B-cells | 1.42 | 0.129 | 0.194 | 1.13 | 0.628 | 0.783 | 0.87 | 0.543 | 0.899 | 1.24 | 0.335 | 0.759 | 1.39 | 0.219 | 0.394 |
| marginal-zone B-cells | 1.61 | 0.044 | 0.164 | 0.74 | 0.237 | 0.783 | 1.14 | 0.575 | 0.899 | 2.26 | 0.0004 | 0.0065 | 0.89 | 0.658 | 0.685 |
| non-switched memory B-cells | 1.65 | 0.078 | 0.164 | 0.89 | 0.696 | 0.783 | 1.28 | 0.390 | 0.781 | 1.49 | 0.147 | 0.759 | 1.26 | 0.478 | 0.574 |
| switched B-cells | 2.35 | 0.0009 | 0.018 | 1.20 | 0.508 | 0.783 | 0.97 | 0.903 | 0.997 | 1.10 | 0.692 | 0.890 | 1.31 | 0.356 | 0.499 |
| plasmablasts | 1.49 | 0.191 | 0.246 | 0.83 | 0.563 | 0.783 | 0.65 | 0.158 | 0.550 | 1.42 | 0.232 | 0.759 | 1.56 | 0.204 | 0.394 |
| transitional B-cells | 1.43 | 0.258 | 0.290 | 1.16 | 0.673 | 0.783 | 0.92 | 0.804 | 0.997 | 1.55 | 0.154 | 0.759 | 1.45 | 0.308 | 0.499 |
| CD4 CM T-cells | 1.31 | 0.057 | 0.164 | 0.89 | 0.431 | 0.783 | 0.95 | 0.743 | 0.997 | 0.98 | 0.859 | 0.935 | 1.49 | 0.012 | 0.188 |
| CD4 EM T-cells | 1.43 | 0.067 | 0.164 | 1.23 | 0.325 | 0.783 | 0.90 | 0.600 | 0.899 | 0.83 | 0.332 | 0.759 | 1.62 | 0.031 | 0.188 |
| naive CD4 T-cells | 1.34 | 0.103 | 0.186 | 0.69 | 0.057 | 0.342 | 0.61 | 0.008 | 0.073 | 1.01 | 0.971 | 0.971 | 1.32 | 0.183 | 0.394 |
| CD4 TEMRA T-cells | 1.71 | 0.164 | 0.227 | 5.79 | 0.00003 | 0.0003 | 1.04 | 0.930 | 0.997 | 0.85 | 0.666 | 0.890 | 1.20 | 0.685 | 0.685 |
| CD8 CM T-cells | 1.24 | 0.295 | 0.313 | 1.16 | 0.499 | 0.783 | 0.81 | 0.326 | 0.734 | 0.97 | 0.883 | 0.935 | 1.69 | 0.026 | 0.188 |
| CD8 EM T-cells | 1.36 | 0.226 | 0.271 | 1.21 | 0.484 | 0.783 | 0.70 | 0.165 | 0.551 | 0.87 | 0.570 | 0.880 | 1.70 | 0.071 | 0.259 |
| naive CD8 T-cells | 1.55 | 0.013 | 0.118 | 1.21 | 0.314 | 0.783 | 0.34 | 0.0000 | 0.0000001 | 0.91 | 0.587 | 0.880 | 1.12 | 0.571 | 0.642 |
| CD8 TEMRA T-cells | 1.44 | 0.114 | 0.186 | 3.01 | 0.00001 | 0.0002 | 1.00 | 0.999 | 0.999 | 1.08 | 0.746 | 0.895 | 1.24 | 0.416 | 0.535 |

**Supplementary Table 9. Results of linear models with log-transformed outcomes showing the association between clinical variables and the composition of peripheral blood immune cell subsets**.

| **Outcome** | **Smoking** | | | **CMV seropositivity** | | | **Age (scale of 30 years)** | | | **Dialysis** | | | **ASCVD** | | |
| --- | --- | --- | --- | --- | --- | --- | --- | --- | --- | --- | --- | --- | --- | --- | --- |
|  | **logFC** | **p** | **adj. P** | **logFC** | **p** | **adj. P** | **logFC** | **p** | **adj. P** | **logFC** | **p** | **adj. P** | **logFC** | **p** | **adj. P** |
| mDC1 | 0.371 | 0.095 | 0.190 | -0.320 | 0.183 | 0.793 | -0.289 | 0.200 | 0.599 | 0.199 | 0.354 | 0.797 | 0.363 | 0.153 | 0.394 |
| mDC2 | -0.414 | 0.055 | 0.190 | 0.001 | 0.998 | 0.998 | 0.229 | 0.294 | 0.755 | 0.127 | 0.540 | 0.898 | 0.426 | 0.084 | 0.302 |
| mDC3 | 0.109 | 0.661 | 0.661 | -0.071 | 0.793 | 0.840 | -0.475 | 0.062 | 0.374 | 0.299 | 0.216 | 0.797 | 0.450 | 0.116 | 0.348 |
| pDC | 0.407 | 0.074 | 0.190 | 0.110 | 0.655 | 0.793 | 0.016 | 0.943 | 0.999 | -0.161 | 0.464 | 0.898 | -0.229 | 0.377 | 0.522 |
| naive B-cells | 0.352 | 0.144 | 0.216 | 0.122 | 0.640 | 0.793 | -0.144 | 0.557 | 0.917 | 0.218 | 0.351 | 0.797 | 0.327 | 0.236 | 0.424 |
| marginal-zone B-cells | 0.475 | 0.053 | 0.190 | -0.302 | 0.254 | 0.793 | 0.134 | 0.587 | 0.917 | 0.815 | 0.0008 | 0.014 | -0.119 | 0.668 | 0.694 |
| non-switched memory B-cells | 0.499 | 0.091 | 0.190 | -0.120 | 0.705 | 0.793 | 0.248 | 0.406 | 0.813 | 0.400 | 0.162 | 0.797 | 0.230 | 0.493 | 0.592 |
| switched B-cells | 0.853 | 0.0019 | 0.033 | 0.185 | 0.522 | 0.793 | -0.032 | 0.906 | 0.999 | 0.099 | 0.702 | 0.902 | 0.273 | 0.372 | 0.522 |
| plasmablasts | 0.397 | 0.208 | 0.267 | -0.190 | 0.576 | 0.793 | -0.436 | 0.174 | 0.599 | 0.353 | 0.249 | 0.797 | 0.442 | 0.221 | 0.424 |
| transitional B-cells | 0.357 | 0.275 | 0.309 | 0.144 | 0.683 | 0.793 | -0.080 | 0.810 | 0.999 | 0.437 | 0.170 | 0.797 | 0.368 | 0.325 | 0.522 |
| CD4 CM T-cells | 0.266 | 0.067 | 0.190 | -0.119 | 0.447 | 0.793 | -0.047 | 0.751 | 0.999 | -0.024 | 0.863 | 0.939 | 0.401 | 0.017 | 0.236 |
| CD4 EM T-cells | 0.360 | 0.079 | 0.190 | 0.210 | 0.342 | 0.793 | -0.105 | 0.612 | 0.917 | -0.185 | 0.349 | 0.797 | 0.484 | 0.039 | 0.236 |
| naive CD4 T-cells | 0.296 | 0.117 | 0.210 | -0.374 | 0.068 | 0.407 | -0.488 | 0.012 | 0.106 | 0.006 | 0.972 | 0.972 | 0.277 | 0.200 | 0.424 |
| CD4 TEMRA T-cells | 0.539 | 0.180 | 0.249 | 1.756 | 0.0001 | 0.0008 | 0.035 | 0.932 | 0.999 | -0.162 | 0.676 | 0.902 | 0.180 | 0.694 | 0.694 |
| CD8 CM T-cells | 0.215 | 0.312 | 0.331 | 0.151 | 0.513 | 0.793 | -0.205 | 0.343 | 0.772 | -0.029 | 0.887 | 0.939 | 0.525 | 0.033 | 0.236 |
| CD8 EM T-cells | 0.311 | 0.242 | 0.291 | 0.194 | 0.499 | 0.793 | -0.362 | 0.181 | 0.599 | -0.141 | 0.583 | 0.898 | 0.531 | 0.082 | 0.302 |
| naive CD8 T-cells | 0.438 | 0.018 | 0.162 | 0.193 | 0.331 | 0.793 | -1.065 | 0.0000001 | 0.000002 | -0.093 | 0.599 | 0.898 | 0.115 | 0.584 | 0.657 |
| CD8 TEMRA T-cells | 0.366 | 0.128 | 0.210 | 1.101 | 0.00005 | 0.0009 | -0.0004 | 0.999 | 0.999 | 0.073 | 0.754 | 0.905 | 0.216 | 0.432 | 0.556 |


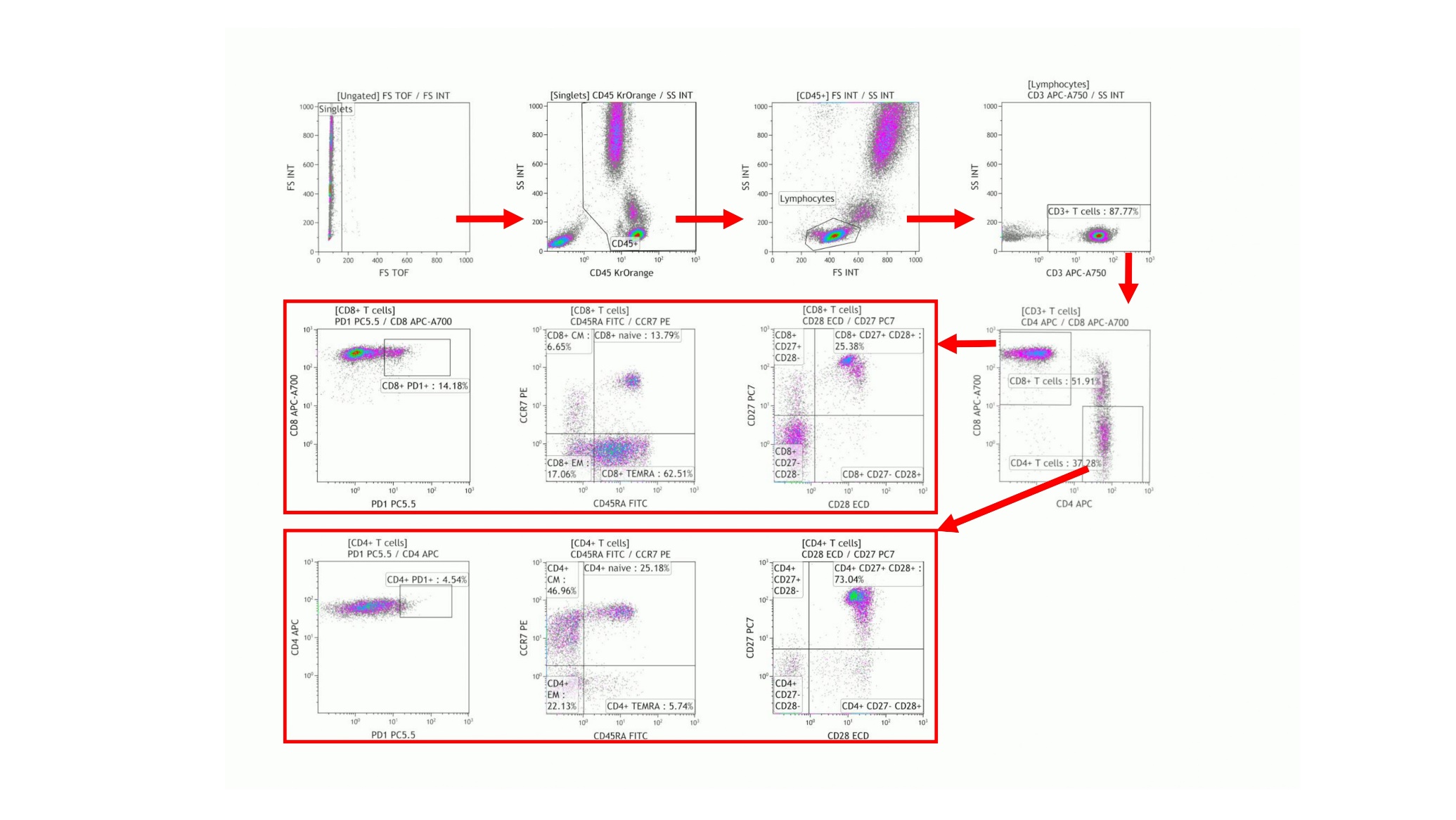
**Supplementary Figure 1. Gating strategy of T cell subpopulations in peripheral blood.**


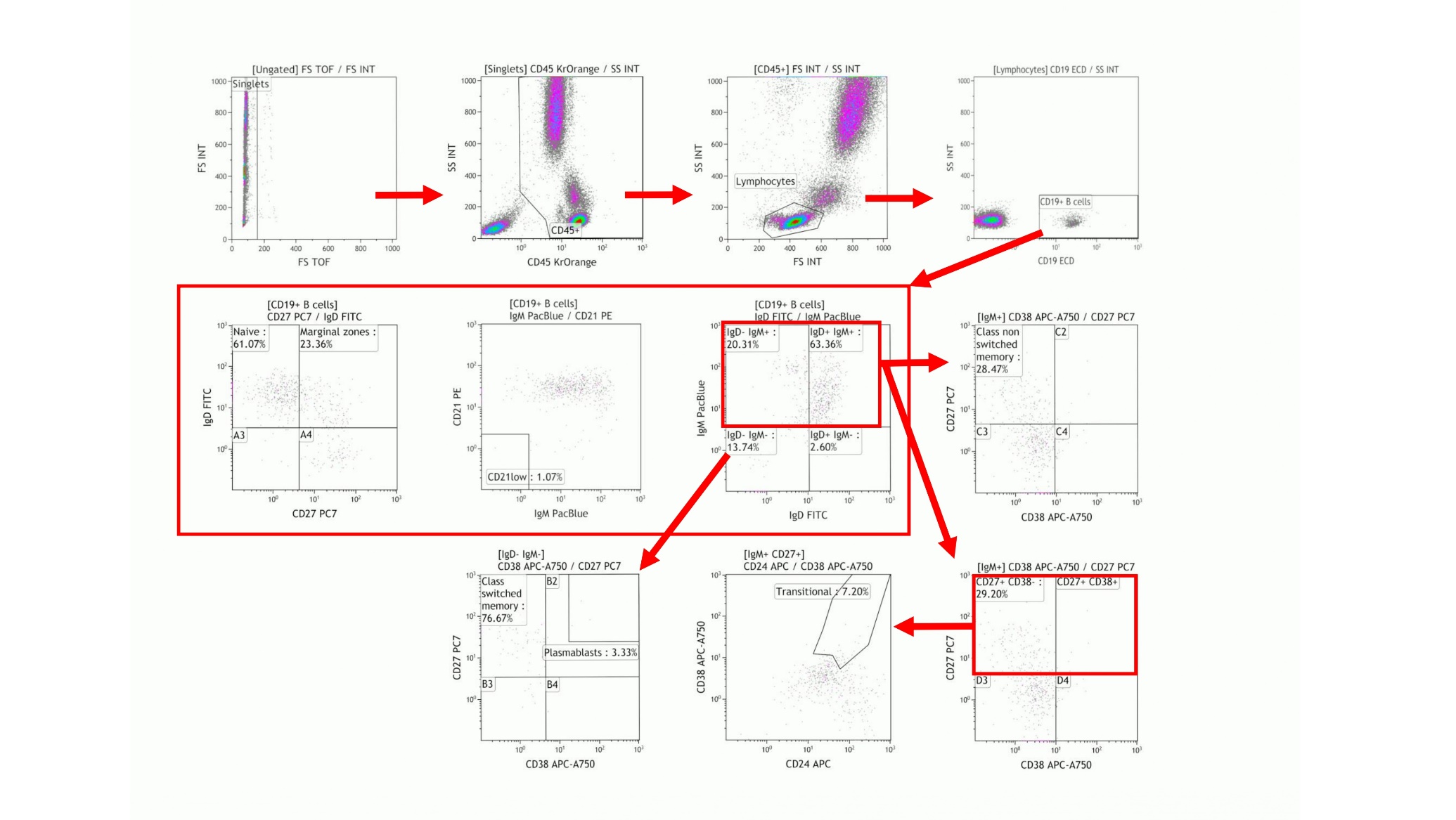
**Supplementary Figure 2. Gating strategy of B cell subpopulations in peripheral blood.**


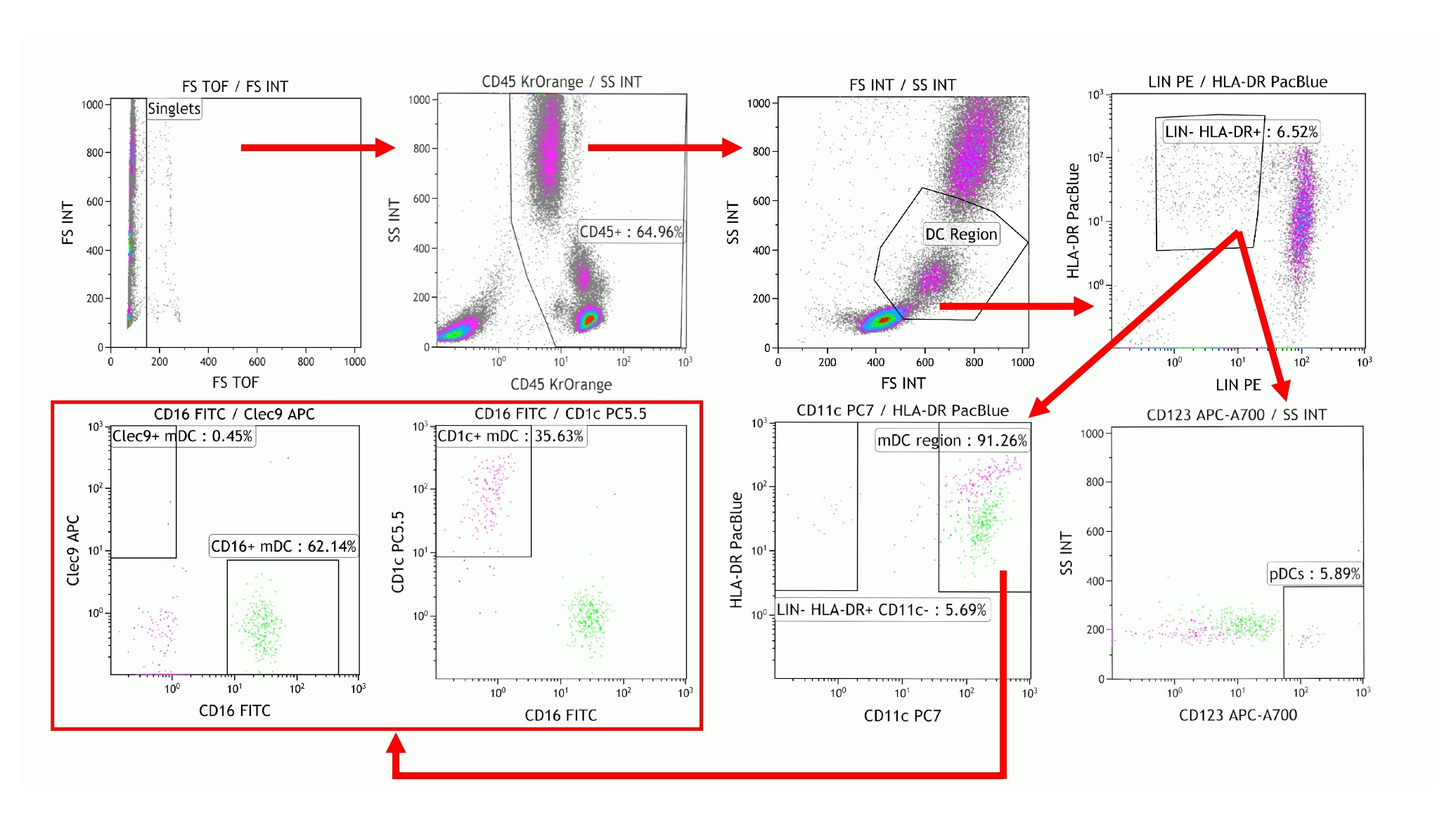
**Supplementary Figure 3. Gating strategy of dendritic cell subpopulations in peripheral blood.**
